# Special Section: Technical Infrastructures, Transnational Protest Movements and the Use of Counter-Expertise

**DOI:** 10.1007/s00048-022-00339-6

**Published:** 2022-08-02

**Authors:** Maria Buck, Kira J. Schmidt

**Affiliations:** 1 Institut für Geschichtswissenschaften und Europäische Ethnologie, Kernfach Wirtschafts- und Sozialgeschichte, Universität Innsbruck, Innrain 52d, 6020 Innsbruck, Austria; 2 Rachel Carson Center for Environment and Society (RCC), Ludwig-Maximilians-Universität, Leopoldstr. 11a, 80802 München, Germany

**Keywords:** Infrastructure, Protest movement, Expertise, Knowledge, Social movement

In February 1997, an international conference on Alpine transit called “Counting on Nature” (*Mit der Natur rechnen*) took place in Innsbruck. The conference was organized by the *Transitforum Austria Tirol*, a citizens’ initiative that actively opposes the increase in transit traffic through the Alps in Tyrol over the last decades. In cooperation with Alpine and nature conservation associations and numerous local citizens’ initiatives, the organizers intended for the event to serve as a platform for the exchange of experience and knowledge. It was also meant to be a sign of transnational resistance of Alpine people against the impact of transit traffic, which they deemed longer acceptable for humans and the environment. The transit conference was part of a protest movement took shape independently throughout the Alpine regions. In terms of transport, the Alps had long formed a natural obstacle to trans-European North-South and East-West traffic, even in the twentieth century. High-speed traffic on rail and road was concentrated along a few corridors like the Brenner Pass between Austria and Italy and the Gotthard Pass between Switzerland and Italy. In response to increasing transit traffic, citizens’ initiatives emerged along these routes. The issue of transit traffic has dominated much of the political and environmental discourse in Tyrol and other Alpine regions since the 1970s in the “age of ecology” (Radkau 2014). Stakeholders recognized the need for transnational cooperation across the Alps and sought to exchange ideas and promote collaboration. Next to “traditional” forms of protest, the actors relied on counter-expertise. They launched their own scientific studies on the effects of heavy traffic on environmental and human health, which were subsequently incorporated into political decision-making processes. The production of knowledge became a central element in the protest movement against transalpine transit traffic.

Exploring these negotiation processes around Alpine transport infrastructure is the objective of the transnational collaborative project “Issues with Europe.” The resistance against transit traffic in the Alps is only one example of a protest movement that drew on transnational connections and used counter-expertise to reach its goals. To better understand these dimensions of protest movements, “Issues with Europe” and the Rachel Carson Center organized a workshop at the Deutsches Museum in Munich from December 12–13, 2019. This special section brings together four contributions from this workshop on “European Infrastructures and Transnational Protest Movements” that place the use of counter-expertise and the transnational dimensions of protest movements against technological infrastructures at the forefront. The authors are particularly interested in the intersection of these dimensions: they set out to explore the transnational dimensions of the circulation of knowledge and expertise within protest movements. To approach this interconnection, the special section addresses the following questions: What role does counter-expertise play in protest movements against technical infrastructures, and under which circumstances is it mobilized by activists? How can counter-expertise shape infrastructures and large-scale technologies and open up the decision-making process to infrastructure users? What role do networks and transnational exchange play for protest movements and the establishment of counter-expertise? What do different actors expect to gain from transnational protest? And, fundamentally: Who are the actors in the production of counter-expertise?

Following the German sociologist Dieter Rucht, we define protest broadly as a “collective, public action by non-state actors that expresses criticism or objection and is linked to the formulation of a social or political concern” (Rucht 2001: 19). Social movements are significant components of participation in society and constitute an important tool that allows democratic societies to integrate minority demands into public discourse (Beyer & Schnabel 2017: 11). In the course of this process of negotiation, protest has come to be regarded as endogenous to society and *not* as an external influence. Protest does not function as a single force, but rather as a resonating sphere of social change (Gassert 2018: 273). Protest against infrastructure projects and large-scale technologies that are already built or still in the planning phase is a common phenomenon. In fact, technology and protest are inextricably linked. This holds especially true for technologies that take on the function of infrastructures (van Laak 2001). Energy and transport infrastructures, in particular, have led to fierce and at times violent protest movements (Nelkin 1975) as will be exemplified by the articles in this special section.

Protest against infrastructure projects and large-scale technologies can be traced back to the nineteenth century (Hasenöhrl 2011: 38–45; Meiske 2021: 259–274), but picked up speed in connection with the new social movements that emerged in several Western European nations over the course of the 1960s and 1970s. These were often catalyzed by opposition to infrastructures which were perceived as symbols for technocratic and authoritarian policies. The rapid expansion of nuclear energy, in particular, served as a hotbed for new social movements, from protestant lay movements to left-wing factions.

Protest against infrastructure has become synonymous with civil disobedience against state and corporate interests—protests against what political scientist and anthropologist James Scott sees as an emblem of “high modernism” in the twentieth century (Scott 1998). Since the 1980s, scholarship in the social sciences and the history of technology has explored the societal negotiation processes inherent to technical infrastructures (Hughes 1983), arguing for technologies’ social co-construction (Bijker et al. 1987) and calling attention to certain truly “public technologies,” like nuclear power, which shape the public while also being shaped by the public (Trischler & Bud 2018). At their core, these societal negotiation processes about infrastructures are negotiations about power: the interrelationship of power and infrastructure is materialized and deposited in the built infrastructure (van Laak 2004; Engels & Schenk 2015). Accordingly, infrastructures can be described as both “products of time” as well as “producers of time” that create path dependencies (Engels 2020: 69; van Laak 2018: 203). Recent scholarship has also explored the epistemic side of infrastructures, framing infrastructures as “places of knowledge production” (Heine & Meiske 2022; Güttler 2020). This approach has also informed this special section.

The transnational dimension of protest movements has been prominently explored in the case of the anti-nuclear movement (Engels 2006; Meyer 2014; Milder 2014), where it appears in a twofold manner. First, anti-nuclear protest emerged all over Europe, making it a transnational issue. Second, actors actively sought out transnational exchange. In the beginning, such protest movements had difficulties becoming international, but during the 1970s and 1980s, global resistance against nuclear power facilitated the transfer of knowledge, the transnational exchange of counter-expertise, and the rise of border-crossing protest cultures.

This transnational dimension of protest constitutes the first focus of this special section. Investigating its history presents a great challenge due to the broad spectrum of exchanges that occur, as well as due to the at times marginal scope (Tompkins 2016: 118; Kirchhof & Meyer 2014). To include all “categories of transnational engagement” (Tompkins 2016: 117), we follow Pierre-Yves Saunier, who proposes an open and broad definition of a transnational perspective, looking at “movements and forces that cut across national boundaries,” i.e. “goods, […] people, […] ideas, words, capital, might, and institutions” (Saunier 2006: 119). In the history of technology, the transnational turn has led to a productive research landscape (van der Vleuten 2008), as exemplified by the “Tensions of Europe” network’s research on the “hidden integration” of Europe (Misa & Schot 2005).

Since the 1990s the rise and fall of expertise and the figure of the expert have gained prominence in both contemporary history and the history of technology (Kohlrausch & Trischler 2014). In broader terms, this relates to the inquiry into the relationship between science and politics (Ash 2010; Fisch & Rudloff 2004; Weingart 2001; Weingart & Lentsch 2008; Oppenheimer et al. 2019), the formation of the “knowledge society” (Böhme & Stehr 1986; Raphael 1996), and questions of legitimization, democratization, and circulation of different types of knowledge and power (Elshakry 2010). In doing so, historical research has drawn on a rich body of research from cultural anthropology, sociology of knowledge, and STS (Reinecke 2010: 3). Recent scholarship tends to emphasize the relational character of expertise, tracing how it serves a concrete need and is produced on request (Grundmann 2017: 26). The fact that expertise is not only to be found in academic and professional circles has been demonstrated by Brian Wynne in his well-known study on nuclear fallout and Cumbrian sheep. He emphasized the “fluidity, porosity and constructedness of the boundaries” (Wynne 1996: 62) between science and lay knowledge.

Precisely this lay expertise has proven to be an effective means of protest movements, taking on the role of counter-expertise (Rucht 1988). The German terms “Gegenwissen” and “Gegenwissenschaft” have been traced back to the protests of 1968, becoming prevalent around 1980 (Stadler et al. 2020). While it has to be said that expertise and counter-competence are just two variants of specialized knowledge, expertise has attracted more scholarly attention. This epistemic dimension of social movements and protest movements—which have often been wrongly accused of hostility towards science (Stadler et al. 2020)—has often been overlooked (Güttler 2019; Topçu 2008). At times, counter-expertise, or rather “alternative science,” has also been scrutinized as a crutch for the interference of big business and U.S. governments in research (Oreskes & Conway 2010).

The contributions gathered here instead approach counter-expertise from a bottom-up perspective, exploring the production of knowledge by concerned citizens or lay people (sometimes in cooperation with recognized experts and official agencies) that has been instrumentalized by various social groups. As will be shown, counter-expertise maintains a central position and fulfills various functions in different protest movements. For example, it is used to mobilize activists, influence political agenda-setting processes, and provide opportunities to interact with “real” experts. The participation of scholars and scientists in these protest movements, i.e. on both “sides,” once again shows how obsolete the demarcation between “official” expertise and lay expertise proves to be in context. The use of counter-expertise in protest movements also testifies to actors’ efforts to solve their problems. It serves as a tool for the protesters to work constructively to achieve their goals and find alternatives. By incorporating counter-expertise as an important element in a protest culture, the protest loses the aura of simply being oppositional. On the contrary, counter-expertise can generate specialized knowledge and actively contribute to the co-production of science and technology: one needs only to think of the efforts involved in collecting data, conducting experiments and taking surveys to challenge official expert opinions. The link between counter-expertise and citizen science has most recently been exemplified for the anti-nuclear movement (Herran 2022).

This special section brings together four case studies of protest movements against energy and transport infrastructures in (Western) Europe—yet these are not the familiar cases of nuclear energy or modes of transportation. Instead, we present cases on shale gas and wind energy, bicycles, and cruise ships. To account for the plurality of topics and regions, this special section combines conceptual approaches from different fields of scholarship. The history of technology, history of science, STS, and cultural anthropology provide a multi-disciplinary and multi-perspectival take on the topic. However, all papers provide an actor-centered perspective by focusing on individual and collective human actions and activities. In the following, we will briefly show the cross-connections between the contributions of the special section.

The first two historical papers focus on how activists—in these cases users of infrastructure—participate in shaping socio-technical systems based on bottom-up production of knowledge. Henk-Jan Dekker takes a look at bicycle protests in the Netherlands—as part of a worldwide movement—to demonstrate how the user’s unique perspective can help shape local policies through the bottom-up production of knowledge. Similarly, Jaume Valentines-Álvarez examines how Catalan activists, taking up global debates on energy and technology in the “Transición” period, transformed Spain’s energy landscape by educating themselves, establishing academic networks, and publishing about wind energy. Both movements from the 1970s share elements of counterculture, criticism of capitalism, and an underlying concern for the environment. These dimensions of protest culture are also present in the two case studies that are located in the present.

Yet in the more contemporary cases, the communication means of the protest groups have received an update. While in the case of the Dutch cyclists and the Catalonian windmill activists print media was the medium of choice to communicate to wider publics at both the national and international levels, today the internet provides the dominant medium. Information and communication technology allowed anti-shale gas activists all over Europe to connect, organize, and exchange scientific information, as Roberto Cantoni shows. His comparative study highlights how different political and social contexts affect activism. Additionally, in another case a mailing list serves as the main mode of communication for the *NoGrandiNavi* activists, who protest against the detrimental impacts of the cruise ship industry on the metropolitan area of Venice in Janine Schemmer’s study. By casting the cruise ship as the ultimate symbol of capitalism, this heterogenous group manages to connect with audiences in media, academia, and various protest movements.

What becomes apparent in studying these protest movements from the Netherlands, Spain, Italy, Poland, and France is the influence of political and social settings on the possibilities and potential successes of counter-expertise as a means of protest. The individual case studies emphasize the opportunity structures of the actors, which offer a defined space for action within which they can acquire, transmit and apply counter-expertise. Similar patterns of social protest that oscillate between the regional and national poles on the one side, and transnational concerns on the other, can be observed in all cases. The transnational dimension of protest is reflected in the circulation and exchange of knowledge and ideas across local and national boundaries.

## Acknowledgements

The authors would like to thank Helmuth Trischler and Martin Meiske for their comments on an earlier version of this article.

The research for this article was funded as a *Lead-Agency-Project* by the research funds of Austria (*Leading House; FWF-Nr. I 3697-G28*), Germany (*DFG-Nr. 392198021*), and Switzerland (*SNF-Nr. 100019E_176479*). For further information visit the project’s website: https://www.uibk.ac.at/projects/issues-with-europe/index.html.en.
